# High *TWIST1 *mRNA expression is associated with poor prognosis in lymph node-negative and estrogen receptor-positive human breast cancer and is co-expressed with stromal as well as ECM related genes

**DOI:** 10.1186/bcr3317

**Published:** 2012-09-11

**Authors:** Muhammad Riaz, Anieta M Sieuwerts, Maxime P Look, Mieke A Timmermans, Marcel Smid, John A Foekens, John WM Martens

**Affiliations:** 1 Erasmus University Medical Center - Daniel den Hoed Cancer Center, Department of Medical Oncology and Cancer Genomics Center, Rotterdam, The Netherlands

**Keywords:** *TWIST1*, Breast cancer, mRNA expression, Prognosis, Nodal status, Estrogen receptor, Metastasis-free survival, Stroma

## Abstract

**Introduction:**

The TWIST homolog 1 (*TWIST1*) is a transcription factor that induces epithelial to mesenchymal transition (EMT), a key process in metastasis. The purpose of this study was to investigate whether *TWIST1 *expression predicts disease progression in a large breast cancer cohort with long-term clinical follow-up, and to reveal the biology related to *TWIST1 *mediated disease progression.

**Methods:**

*TWIST1 *mRNA expression level was analyzed by quantitative real-time reverse polymerase chain reaction (RT-PCR) in 1,427 primary breast cancers. In uni- and multivariate analysis using Cox regression, *TWIST1 *mRNA expression level was associated with metastasis-free survival (MFS), disease-free survival (DFS) and overall survival (OS). Separate analyses in lymph node-negative patients (LNN, n = 778) who did not receive adjuvant systemic therapy, before and after stratification into estrogen receptor (ER)-positive (n = 552) and ER-negative (n = 226) disease, were also performed. The association of *TWIST1 *mRNA with survival endpoints was assessed using Kaplan-Meier analysis. Using gene expression arrays, genes showing a significant Spearman rank correlation with *TWIST1 *were used to identify overrepresented Gene Ontology (GO) terms and Kyoto Encyclopedia of Genes and Genomes (KEGG)-annotated biological pathways.

**Results:**

Increased mRNA expression level of *TWIST1 *analyzed as a continuous variable in both uni- and multivariate analysis was associated with shorter MFS in all patients (hazard ratio (HR): 1.17, 95% confidence interval, (95% CI):1.09 to 1.26; and HR: 1.17, 95% CI: 1.08 to 1.26; respectively), in LNN patients (HR: 1.22, 95% CI: 1.09 to 1.36; and HR: 1.21, 95% CI: 1.07 to 1.36; respectively) and in the ER-positive subgroup of LNN patients (HR: 1.34, 95% CI: 1.17 to 1.53; and HR: 1.32, 95% CI: 1.14 to 1.53; respectively). Similarly, high *TWIST1 *expression was associated with shorter DFS and OS in all patients and in the LNN/ER-positive subgroup. In contrast, no association of *TWIST1 *mRNA expression with MFS, DFS or OS was observed in ER-negative patients. Genes highly correlated with *TWIST1 *were significantly enriched for cell adhesion and ECM-related signaling pathways. Furthermore, *TWIST1 *mRNA was highly expressed in tumor stroma and positively related to tumor stromal content (*P *<0.001).

**Conclusions:**

*TWIST1 *mRNA expression is an independent prognostic factor for poor prognosis in LNN/ER-positive breast cancer. The biological associations suggest an involvement of the tumor microenvironment in *TWIST1*'s adverse role in breast cancer.

## Introduction

Breast cancer is one of the most frequently diagnosed cancers and the leading cause of cancer related deaths among females of the Western world [1]. Patients do not die from the primary tumor, but from metastases, which already are resistant or acquire resistance to systemic therapy. Metastasis is a complex, multi-step process in which malignant cells undergo sequential molecular changes helping them to disengage from primary sites, intravasate into blood vessels, extravasate to distant organs and finally colonize secondary sites. Each of these metastatic steps is affected by aberrant expression of a variety of transcription factors and among them, TWIST homologue 1 *(TWIST1) *is considered an important regulator of disease progression [2].

The TWIST1 protein, encoded by the *TWIST1 *gene, is a member of a large protein family called basic helix-loop-helix (bHLH) transcription factors [3]. Most family members contain a bHLH domain, which enables it to target specific DNA sequences and thereby allowing them to regulate developmental processes in many organs and tissues. *TWIST1 *plays a key role in the regulation of embryogenesis, gastrulation and mesoderm formation during early embryonic development of Drosophila and many other species [4,5]. An autosomal mutation pattern in the *TWIST1 *gene leads to Saethre-Chotzen syndrome, a genetic condition characterized by premature fusion of skull bones affecting symmetrical growth of the head and face [6]. In children, TWIST1 protein is involved in adequate maturation of the skull and spine bones and normal development of arms and hind legs.

More recently, TWIST1 protein has been implicated in various carcinomas, including breast cancer, where it plays a role in metastasis through activation of a biologically latent developmental process called epithelial to mesenchymal transition (EMT) [7,8]. In the EMT process, malignant epithelial cells undergo cytoskeletal changes, including the down-regulation of epithelial markers, such as E-cadherin and co-expressed catenins and up-regulation of mesenchymal markers, such as vimentin, N-cadherin and fibronectin. EMT transformed malignant cells are more motile and can be more efficient in invading the surrounding tissues and as a result metastasize to distant organs [9].

In this large retrospective study of 1,427 primary breast cancer patients, we determined whether the *TWIST1 *gene expression level is a prognostic marker. To avoid possible confounding effects of therapy and to study the natural course of the disease, we particularly focused on the subgroup of 778 lymph node-negative (LNN) patients who did not receive any adjuvant systemic therapy. Additionally, to understand the biological context of TWIST1, we have identified, using available Affymetrix U133A gene expression data [10,11], genes and biological pathways co-expressed with *TWIST1*. By doing this, we identified a clear link between the tumor microenvironment and *TWIST1 *expression in clinical breast cancer.

## Methods

### Patients

**Figure 1:**
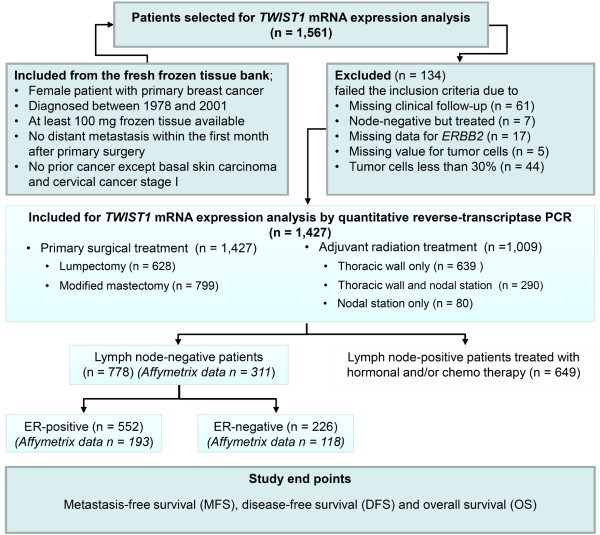
**Study design, patient inclusion criteria and patient sub-cohorts analyzed**. Associations of *TWIST1 *mRNA expression levels with tumor aggressiveness were evaluated in all patients and in LNN, LNN/ER-positive and LNN/ER-negative patients. The LNN patients in this study did not receive adjuvant systemic therapy.

This study was approved by the institutional medical ethics committee of the Erasmus MC (MEC 02.953). The study was performed in accordance with the Code of Conduct of the Federation of Medical Scientific Societies in The Netherlands [12]; informed consent was not required. Wherever possible, the study has been reported in line with the Reporting Recommendations for Tumor Marker Prognostic Studies guidelines [13,14]. Breast cancer tissue specimens from 1,427 female patients with primary operable breast cancer were included in the study. Tumor samples were originally submitted to our reference laboratory from 25 regional hospitals for biochemical assessment of steroid hormone receptor status. Guidelines for primary treatment were similar in all hospitals. All available frozen breast tumor samples of at least 100 mg from female patients with invasive breast cancer who entered the clinic during the period of 1978 to 2001, and from whom detailed clinical follow-up data were available were processed. Moreover, these patients had no distant metastasis within the first month after primary surgery and no prior cancer except basal skin carcinoma and cervical cancer stage I. ER, PGR and HER2 (ERBB2) levels were assessed by quantitative real-time reverse transcriptase PCR (qRT-PCR) as described before [15-17]. Lymph node involvement, tumor size and grade were extracted from the pathology reports as obtained from the hospitals. Primary surgical treatment was lumpectomy in 628 patients (44%) or modified mastectomy for 799 patients (56%). One thousand and nine patients (71%) received adjuvant radiotherapy at the thoracic wall only (n = 639) and either at the thoracic wall and nodal stations (n = 290) or at the nodal stations only (n = 80). Thirty-three percent of the patients had T1 tumors. The median age of the patients at surgery was 55 years (range, 23 to 89 years). Routine post-surgical follow-up and the definition of the endpoints of MFS, DFS and OS were described before [11,18]. During follow-up, a local/regional relapse for 127 patients was not counted as an event in the analysis of MFS. The median follow-up time was 104 months (range, 4 to 262 months) with 707 and 669 events in the analyses of MFS and OS, respectively. Study design, patient inclusion criteria and patient subgroups included in the analyses are given in Figure 1. Other relevant clinico-pathological characteristics are listed in Table 1.

**Table 1:** Association of *TWIST1 *mRNA expression with standard clinical and pathological characteristics of patients and tumors

Characteristics	No. patients	*TWIST1 *mRNA expression (Median value*)	*P*-value
**Age (years)**			
<40	188	0.025	
41 to 55	530	0.028	
56 to 70	471	0.030	
>70	238	0.020	0.04^†^
**Menopausal status**			
Premenopausal	603	0.027	
Postmenopausal	824	0.027	0.21^‡^
**Tumor size**			
pT1, <2 cm	463	0.031	
pT2, >2 to <5	807	0.025	
pT3, >5 + pT4	157	0.028	0.009^¶^
**Lymph nodes involved**			
0	778	0.026	
1 to 3	288	0.025	
>3	361	0.030	0.007^¶^
**Grade**			
Poor	800	0.026	
Good/Moderate	230	0.027	
Unknown	397	0.029	0.47^¶^
**Tumor histology**			
IDC	957	0.027	
ILC	112	0.044	<0.001^‡, §^
Medullary	31	0.016	
Mucinous	39	0.008	<0.001^¶^
**ER status **(mRNA)			
Negative	360	0.021	
Positive	1,067	0.029	0.06^†^
**PGR status **(mRNA)			
Negative	572	0.023	
Positive	855	0.029	<0.001^†^
**ERBB2 status **(mRNA)			
Negative	1,187	0.026	
Positive	240	0.032	<0.001^†^

Abbreviations: IDC, invasive ductal carcinoma; ILC, invasive lobular carcinoma.

* Expression values after normalization to our set of three reference genes (*B2M*, *HMBS *and *HPRT1*).

† *P *Spearman rank correlation test.

‡ *P *for Mann-Whitney test.

¶ *P *for Kruskal-Wallis test.

§ *TWIST1 *expression was compared between IDC and ILC tumors.

### Tissue processing and estimation of the amount of epithelial tumor cells

Tissue processing was done as described in detail previously [15,19]. In brief, 20 to 60 cryostat sections of 30 µm size corresponding to 30 to 100 mg weight were cut from frozen tissues. Three 5 µm sections were cut before, in between and after cutting the sections for RNA isolation, and were stained with hematoxylin and eosin (H&E) to assess the amount of tumor cells relative to the amount of surrounding stromal cells. The amount of nuclei evidently of epithelial tumor cell origin relative to the amount of surrounding stromal cells was estimated with a 100-fold magnification in 10 different areas covering the area of each of the three H&E sections. Only specimens with at least 30% of the nuclei of epithelial tumor cell origin and distributed uniformly over at least 70% of the section area were included. Like before [16], these estimates were used to dichotomize our tumor cohort at the median level of 70% tumor cell nuclei in stromal-rich (SR) (primary tumors containing >30% stromal components) and stromal-poor (SP) (primary tumors containing at least 70% tumor cells).

### RNA isolation, cDNA synthesis, and quantification of specific mRNA species

RNA isolation, cDNA synthesis, quantification of specific mRNA species and quality control checks were done as described previously by Sieuwerts *et al*. [15]. qRT-PCR was performed in 25-µl reaction volume in a Mx3000P Real-time PCR System (Agilent, Amsterdam, The Netherlands) using a commercially available gene expression assay for *TWIST1 *(Hs00361186_m1) and for *VIM *(Hs Hs00185584_m1) from Applied Biosystems/Life Technologies (Nieuwerkerk aan den IJssel, The Netherlands). For *EPCAM *mRNA measurement, a combination of the following primers and probe was used (F: 3'-AGT TTG CGG ACT GCA CTT CA, R: 3'- AAT ACT CGT GAT AAA TTT TGG ATC CA, FAM-labeled MGB probe: AAG GAG ATC ACA ACG CGT). Primer sequences for estrogen receptor-α (*ESR1*), progesterone receptor (*PGR*), *ERBB2 *and the reference genes (*HMBS*, *HPRT1 *and *B2M*), as well as how PCR reactions and validations were carried out to ensure PCR specificity, were done as described before [15,17]. Levels of mRNA expression were quantified relative to the set of reference genes as described before [15].

### Laser capture microdissection

Laser capture microdissection (LCM) was performed according to a previously published method [20]. Briefly, 8 µm sections of fresh frozen tumor tissue were cryosectioned and pasted on UV sterilized polyethylene naphthalate (PEN) covered glass slides (P.A.L.M. Microlaser Technologies, Bernried, Germany). The slides were air dried for 10 sec at room temperature, fixed in ice-cold 70% ethanol and washed shortly in Milli-Q water. The slides were then stained for 15 sec in Haematoxylin (Klinipath 4085.9005, Duiven, The Netherlands) and washed again in Milli-Q water. Subsequently, the slides were dehydrated twice in RNase-free 50, 70, 95 and 100% ethanol for 30 sec each, and air-dried. Next, LCM was performed directly on the stained sections. From consecutive cryosections of a tumor tissue, both the stromal content and tumor epithelial cells were collected separately in triplicate in P.A.L.M. tube caps containing 25 μl of tissue lysis (RLT+) buffer (Qiagen 1053393, Venlo, The Netherlands) using a P.A.L.M. LCM device, type P-MB (P.A.L.M. Microlaser Technologies AG). Both stromal and epithelial cell fractions were then spun down into 0.5-ml Eppendorf Protein LoBind tubes (Eppendorf, Hamburg, Germany) containing 225 μl RLT+ buffer and used for RNA isolation.

### Immunohistochemistry (IHC)

Sections of 4 µm were cut from formalin-fixed paraffin-embedded tissues, pasted on glass slides and dried overnight at 37°C. The slides were dewaxed in xylene solution and hydrated in a series of different percentages of alcohol (100%, 95%, 75% and 50%). The antigen was retrieved for 40 minutes in a hot water bath (at 95°C ) in Tris-EDTA buffer, pH 9.0 (DAKO; S2367, Glostrup, Denmark) and cooled for 20 minutes at room temperature. After retrieval, slides were immersed in 3% hydrogen peroxide for 10 minutes to block endogenous peroxidase activity, and in a DAKO protein-free serum blocking solution for 30 minutes to block non-specific binding sites. The slides were then incubated overnight at 4°C with a mouse monoclonal primary antibody raised against a human recombinant fragment of TWIST1 (AbCam TWIST2C1a, ab50887, Cambridge, UK) and diluted 1:100 in an antibody diluent (DAKO). Negative controls were made by replacing the primary antibody with mouse immunoglobulin at an appropriate dilution. Next, the slides were treated with a secondary antibody (envision mouse kit, DAKO) and visualized using DAB (envision kit, DAKO). The slides were counterstained with hematoxylin and dehydrated through graded alcohol and xylene. Evaluation of the immunostaining and histoscoring were performed according to a previously published method [21]. Briefly, a histoscore of 0 to 300 was given to each tumor by multiplying the proportion of cells with nuclear staining in a tissue by the intensity of the staining as follows; histoscore = (0 × percentage not stained) + (1 × percentage weakly stained) + (2 × percentage moderately stained) + (3 × percentage strongly stained). The histoscores calculated in this way were correlated with *TWIST1 *mRNA expression. The intensity of TWIST1 stromal expression in tumor tissues was scored as no, weak, moderate or strong staining.

### Identification of *TWIST1 *co-expressed genes on Affymetrix U133A gene-chips and identification of Gene Ontology (GO) terms and biological pathways

In this analysis we used *TWIST1 *mRNA expression, which was previously measured by Affymetrix U133A gene-chips (Affymetrix, Santa Clara, CA, USA) (probe set 213943_at) [10,11,22]. The mRNA expression levels of *TWIST1 *measured on the Affymetrix-array and by qRT-PCR were highly correlated in an overlap of 193 LNN/ER-positive and 118 LNN/ER-negative tumor specimens using the Spearman-Rank correlation test (Spearman rank correlation, Rs = 0.7; *P *<0.001 for both). Genes showing a significant positive or negative correlation with *TWIST1 *were identified by using these Affymetrix-array data after all genes were corrected for multiple testing by applying a false discovery rate (FDR) of 5% [23]. Biological pathways and GO terms from genes significantly co-expressed with *TWIST1 *were identified using the ArrayTrack™software (NCTR/FDA, Jefferson, AR 72079, USA) [24]. The overrepresented pathways and GO terms identified by ArrayTrack™ were based on Fisher's exact statistics. Details about how ArrayTrack™ software functions can be retrieved from the website [25]. The gene expression data used have previously been deposited in the National Center for Biotechnology Information/Gene Expression Omnibus database entries GSE2034 and GSE5327.

### Statistics

Computations were done with the use of the STATA statistical package, release 11.2 (STATA Corp., College Station, TX, USA). Differences in levels were assessed with the Mann-Whitney U test or Kruskal-Wallis test. In these tests, patient and tumor characteristics were used as grouping variables. The strengths of the associations between continuous variables were tested with the Spearman rank correlation test. *TWIST1 *mRNA expression levels measured by qRT-PCR were log transformed to reduce the skewness (Skewness-Kurtosis normality test, *P *<0.05) and to attain symmetric distribution (Additional file 1: Figure S1). The prognostic value of the clinical and biological variables, with MFS, OS and DFS as the end points in the univariate and multivariate analyses, were investigated with the use of the Cox proportional hazard model. The hazard ratio (HR) and its 95% confidence interval (95% CI) were derived from these models by analyzing *TWIST1 *mRNA expression levels as a continuous variable or after dividing their levels into quartiles. The proportionality assumption was investigated with a test based on the Schoenfeld residuals. Kaplan-Meier survival analysis was performed and a log-rank test was used to assess a trend of survivor's function across four quartiles of *TWIST1 *mRNA expression levels. All *P*-values are two-sided, and *P *<0.05 was considered statistically significant.

## Results

### Association of *TWIST1 *mRNA expression with standard clinical and pathological characteristics of patients and tumors

Association of *TWIST1 *mRNA expression levels with clinical and pathological characteristics of patients and tumors are shown in Table 1. Tumor *TWIST1 *mRNA expression was higher in patients with >3 positive lymph nodes, and lower in pT2 tumors and in older patients. Furthermore, *TWIST1 *mRNA expression was higher in invasive lobular carcinoma (ILC) compared with invasive ductal carcinoma (IDC). Mucinous and medullary-type of tumors showed the lowest *TWIST1 *mRNA expression levels. In addition, *TWIST1 *mRNA expression level was higher in *PGR*-positive and *ERBB2*-overexpressing tumors, but was not associated with tumor grade and menopausal status.

### Association of *TWIST1 *mRNA expression with prognosis in primary breast cancer patients

**Figure 2:**
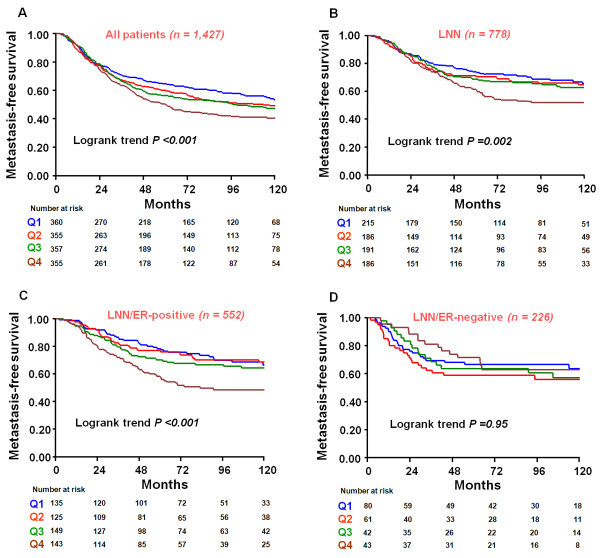
**Kaplan-Meier survival curves presenting the association of *TWIST1 *mRNA expression with metastasis-free survival in: A: all 1,427 patients; **B: **LNN patients only, **C: **LNN and ER-positive patients and **D: **LNN and ER-negative patients**. The LNN patients in this study did not receive adjuvant systemic therapy. The patients are divided into four quartiles (Q1 (low) to Q4 (high)) based on *TWIST1 *mRNA expression levels after normalizing these to the expression of our set of three reference genes (*B2M, HMBS *and *HPRT1*). Patients at risk at various time points are indicated.

To explore the prognostic significance of *TWIST1 *mRNA expression in primary breast cancer patients, we first performed Cox uni- and multivariate analyses (traditional factors in the multivariate analysis were; age, menopausal status, number of positive lymph nodes, tumor size, tumor grade, ER status, PGR status and ERBB2 status) for MFS, DFS and OS as a function of continuous *TWIST1 *mRNA expression levels. In all 1,427 patients, increasing *TWIST1 *mRNA expression levels was associated with shorter MFS in both uni- and multivariate analysis (HR: 1.17, 95% CI: 1.09 to 1.26; *P *<0.001 and HR: 1.17, 95% CI: 1.08 to 1.26; *P *<0.001; respectively) (Table 2). To visualize the prognostic value of *TWIST1 *mRNA expression in Kaplan-Meier survival curves, we divided *TWIST1 *expression levels into four quartiles Q1 (low) to Q4 (high) (Figure 2). To exclude a possible confounding treatment effect, we also evaluated the prognostic value of *TWIST1 *mRNA expression in LNN-patients who did not receive any adjuvant systemic therapy. We also stratified these LNN patients into ER-positive and ER-negative subgroups as these subgroups are considered biologically distinct. In the analysis of all 778 LNN patients, an association of increasing *TWIST1 *mRNA expression levels with shorter MFS was observed in both uni- and multivariate analysis (univariate HR: 1.22, 95% CI: 1.09 to 1.36; *P *= 0.001 and HR: 1.21, 95% CI: 1.07 to 1.36; *P *= 0.001; respectively) (Additional file 2: Table S1). Moreover, within the LNN subgroup of patients, the association of increasing *TWIST1 *mRNA levels with shorter MFS was confined to the ER-positive subgroup of patients (univariate (HR: 1.34, 95% CI: 1.17 to 1.53; *P *<0.001) and multivariate (HR: 1.32, 95% CI: 1.14 to 1.53; *P *<0.001)) (Table 3). There was no association of *TWIST1 *mRNA levels with MFS in the ER-negative subgroup of patients (univariate HR = 1.05, 95% CI: 0.86 to 1.29; *P *= 0.64) (Additional file 2: Table S2). Kaplan-Meier survival curves for all patients and subgroups of patients with LNN and LNN/ER-positive and LNN/ER-negative disease, after dividing these cohorts in quartiles (Q1 to Q4), are shown in Figure 2A-D. Cox regression analysis for MFS showed that in the LNN/ER-positive group the quartile with the highest *TWIST1 *mRNA levels (Q4) had a two-fold increased hazard ratio compared with the quartile with the lowest *TWIST1 *mRNA levels (Q1) (Table 3).

**Table 2:** Model for uni-and multivariate analysis for metastasis-free survival in all patients (n = 1,427)

Factors	No. patients		Univariate			Multivariate	
			
		HR	95% CI	*P*-value	HR	95% CI	*P*-value
**Age (years)**							
<40	188	1			1		
41 to 55	530	0.92	0.73 to 1.16	0.47	0.88	0.69 to 1.12	0.30
56 to 70	471	1.02	0.80 to 1.29	0.88	0.85	0.59 to 1.21	0.36
>70	238	0.80	0.66 to 1.06	0.12	0.69	0.47 to 1.03	0.07
**Menopausal status**							
Premenopausal	603	1			1		
Postmenopausal	824	1.07	0.92 to 1.25	0.35	1.23	0.94 to 1.62	0.14
**Tumor size**							
pT1, <2 cm	463	1			1		
pT2, >2 to <5	807	1.65	1.39 to 1.97	<0.001	1.38	1.15 to 1.65	<0.001
pT3, >5 + pT4	157	2.80	2.20 to 3.58	<0.001	1.80	1.39 to 2.33	<0.001
**Lymph nodes involved**							
0	778	1			1		
1 to 3	288	1.79	1.48 to 2.17	<0.001	1.71	1.41 to 2.08	<0.001
>3	361	2.92	2.46 to 3.46	<0.001	2.63	2.19 to 3.16	<0.001
**Grade**							
Poor	800	1			1		
Good/Moderate	230	0.51	0.40 to 0.65	<0.001	0.64	0.50 to 0.82	<0.001
Unknown	397	0.89	0.75 to 1.05	0.17	0.99	0.83 to 1.17	0.87
**ER status **(mRNA)							
Negative	360	1			1		
Positive	1,067	0.82	0.70 to 0.97	0.02	0.83	0.67 to 1.04	<0.001
**PGR status **(mRNA)							
Negative	572	1			1		
Positive	855	0.75	0.64 to 0.87	<0.001	0.87	0.71 to 1.06	0.17
**ERBB2 status **(mRNA)							
Negative	1,187	1			1		
Positive	240	1.30	1.08 to 1.57	0.006	1.18	0.97 to 1.43	0.09
					**Addition to the base model**		
***TWIST1 *mRNA level^† ^**(Continuous)	1,427	1.17	1.09 to 1.26	<0.001	1.17	1.08 to 1.26	<0.001
***TWIST1 *mRNA level^†^**							
*TWIST1 *quartile 1	360	1			1		
*TWIST1 *quartile 2	355	1.16	0.94 to 1.45	0.17	1.09	0.88 to 1.37	0.43
*TWIST1 *quartile 3	357	1.22	0.98 to 1.51	0.08	1.25	1.00 to 1.56	0.05
*TWIST1 *quartile 4	355	1.49	1.20 to 1.83	<0.001	1.43	1.15 to 1.77	0.001

†*TWIST1 *mRNA expression level (log-transformed after normalization to the expression of three reference genes) was separately added to the base multivariate model that included the factors age, menopausal status, tumor size, grade, lymph-node status, *ER*, *PGR*, and *ERBB2 *mRNA expression levels, tested both as a continuous variable and in four quartiles.

**Table 3:** Model of uni- and multivariate analysis for metastasis-free survival in lymph-node negative and ER-positive patients (n = 552)

Factors	No. patients		Univariate			Multivariate	
			
		HR	95% CI	*P*-value	HR	95% CI	*P*-value
**Age (years)**							
<40	66	1			1		
41 to 55	187	0.97	0.64 to 1.49	0.90	1.05	0.68 to 1.64	0.81
56 to 70	179	0.79	0.51 to 1.23	0.29	0.81	0.40 to 1.66	0.57
>70	120	0.59	0.35 to 0.99	0.05	0.59	0.27 to 1.28	0.18
**Menopausal status**							
Premenopausal	215	1			1		
Postmenopausal	337	0.76	0.57 to 1.00	0.05	0.99	0.56 to 1.74	0.98
**Tumor size**							
pT1, <2 cm	251	1			1		
pT2, >2 to <5	276	1.12	0.84 to 1.49	0.43	1.09	0.82 to 1.46	0.34
pT3, >5 + pT4	25	1.62	0.84 to 3.11	0.15	1.92	0.99 to 3.75	0.06
**Grade**							
Poor	257	1			1		
Good/Moderate	124	0.53	0.35 to 0.79	0.002	0.56	0.37 to 0.85	0.007
Unknown	171	1.06	0.78 to 1.44	0.70	1.20	0.87 to 1.64	0.26
**PGR status **(mRNA)							
Negative	116	1		1			
Positive	436	0.67	0.49 to 0.92	0.013	0.71	0.51 to 0.99	0.04
**ERBB2 status **(mRNA)							
Negative	475	1			1		
Positive	77	1.51	1.06 to 2.15	0.02	1.39	0.97 to 2.00	0.08
					**Addition to the base model**		
***TWIST1 *mRNA level^† ^**(Continuous)	552	1.34	1.17 to 1.53	<0.001	1.32	1.14 to 1.53	<0.001
***TWIST1 *mRNA level^†^**							
*TWIST1 *quartile 1	135	1			1		
*TWIST1 *quartile 2	125	1.02	0.65 to 1.61	0.92	0.90	0.57 to 1.43	0.67
*TWIST1 *quartile 3	149	1.28	0.85 to 1.94	0.24	1.09	0.71 to 1.68	0.70
*TWIST1 *quartile 4	143	2.06	1.39 to 3.04	<0.001	1.87	1.25 to 2.80	0.002

**† ***TWIST1 *mRNA expression level (log-transformed after normalization to the expression of three reference genes) was separately added to base multivariate model that included the factors age, menopausal status, tumor size, grade, *ER*, *PGR*, and *ERBB2 *mRNA expression levels, tested both as a continuous variable and in four quartiles.

We also related *TWIST1 *mRNA expression levels with OS in all patients, in the LNN cases as a whole and after stratifying by ER status. Similarly as observed for MFS, increasing *TWIST1 *mRNA expression levels was associated with shorter OS in all patients (univariate HR: 1.10, 95% CI: 1.08 to 1.26, *P *= 0.013; multivariate HR: 1.11, 95% CI: 1.03 to 1.20, *P *= 0.006), in the LNN (univariate HR: 1.13, 95% CI: 1.01 to 1.27, *P *= 0.03; multivariate HR: 1.16, 95% CI: 1.03 to 1.30, *P *= 0.01) and in the LNN/ER-positive group of patients (univariate HR: 1.26, 95% CI: 1.09 to 1.45, *P *= 0.001; multivariate HR: 1.27, 95% CI: 1.11 to 1.46, *P *= 0.001). These associations were not significant in the LNN/ER-negative group of patients (univariate HR: 0.96, 95% CI: 0.78 to 1.18, *P *= 0.69; multivariate HR: 0.98, 95% CI: 0.79 to 1.21, *P *= 0.85). The results are included in Additional file 2: Tables S3-S6. Kaplan-Meier survival curves showing OS for all patients, and also for the subgroups of patients after dividing them into quartiles based on *TWIST1 *mRNA levels are shown in Additional file 1: Figure S2A-D.

We finally analyzed whether *TWIST1 *mRNA expression levels were associated with DFS in all patients and in LNN/ER-positive patients only, and obtained similar results as were obtained for MFS and OS (Additional file 2: Tables S7-8).

### Investigation of *TWIST1 *related biological pathways in LNN breast cancer

To explore the underlying biology of *TWIST1*-associated disease progression in breast cancer, we determined *TWIST1 *co-expressed genes previously measured on Affymetrix U133A gene-chips [10,11]. Since we found *TWIST1 *to be associated with poor prognosis in LNN/ER-positive patients only, we first decided to include only the LNN/ER-positive samples to examine the *TWIST1 *co-expressed genes. After using a Spearman correlation test followed by applying a multiple testing correction at a FDR of 5%, we found 1,847 genes to be positively correlated and 1,445 genes to be negatively correlated with *TWIST1 *mRNA expression (Additional file 3: Table S9A, B). To reveal which biological pathways might be associated with *TWIST1 *mRNA expression, we performed pathway analyses using the genes co-expressed with *TWIST1 *and the KEGG pathway database as input for the analysis in the ArrayTrack™ software package. The three most significantly overrepresented pathways containing these *TWIST1 *co-expressed genes were: Focal adhesion pathway (Fisher's exact *P *= 1.0 × 10^-8^), ECM-receptor interaction pathway (Fisher's exact *P *= 9.9 × 10^-7^), and TGF-beta signaling pathway (Fisher's exact *P *= 2.6 × 10^-4^) (Additional file 3: Table S10). In addition, GO term enrichments among *TWIST1 *co-expressed genes identified "cell adhesion", "extracellular structure organization" and "anatomical structure formation involved in morphogenesis" as the top three significant GO terms (level >3; gene hits >5; *P-*values <0.001) in the category of "biological processes", and the GO terms, "extracellular matrix" proteinaceous extracellular matrix and "extracellular matrix part" as the top three significant GO terms in the category "protein cellular components" (Additional file 3: Table S11).

To evaluate a possible *TWIST1 *mediated biological difference between LNN/ER-positive and LNN/ER-negative patients, we also performed pathway analyses using *TWIST1 *co-expressed genes (n = 1,141 positively and n = 498 negatively correlated genes with *TWIST1*, respectively, Additional file 3: Table S12A, B) measured on Affymetrix arrays from LNN/ER-negative patients (n = 118). As observed in LNN/ER-positive patients, these analyses identified Focal adhesion and ECM-receptor interaction pathways as the two most significant pathways. But, in contrary to what observed in LNN/ER-positive patients, the TGF-beta signaling pathway was not found to be the third most significant pathway in LNN/ER-negative patients (Additional file 3: Table S13). We also performed pathway analysis using *TWIST1 *co-expressed genes in all LNN patients irrespective of ER status. Analyses revealed only Focal adhesion and ECM-receptor interaction pathways as the two most significant pathways, but the TGF-beta signaling pathway was not found to be the third top significant pathway in all patients (Additional file 3: Table S14). Altogether, these data suggest that the co-expression of the TGF-beta signaling pathway with *TWIST1 *gene is more prominent in LNN/ER-positive cancer.

### Association of *TWIST1 *mRNA expression levels with stromal content

**Figure 3:**
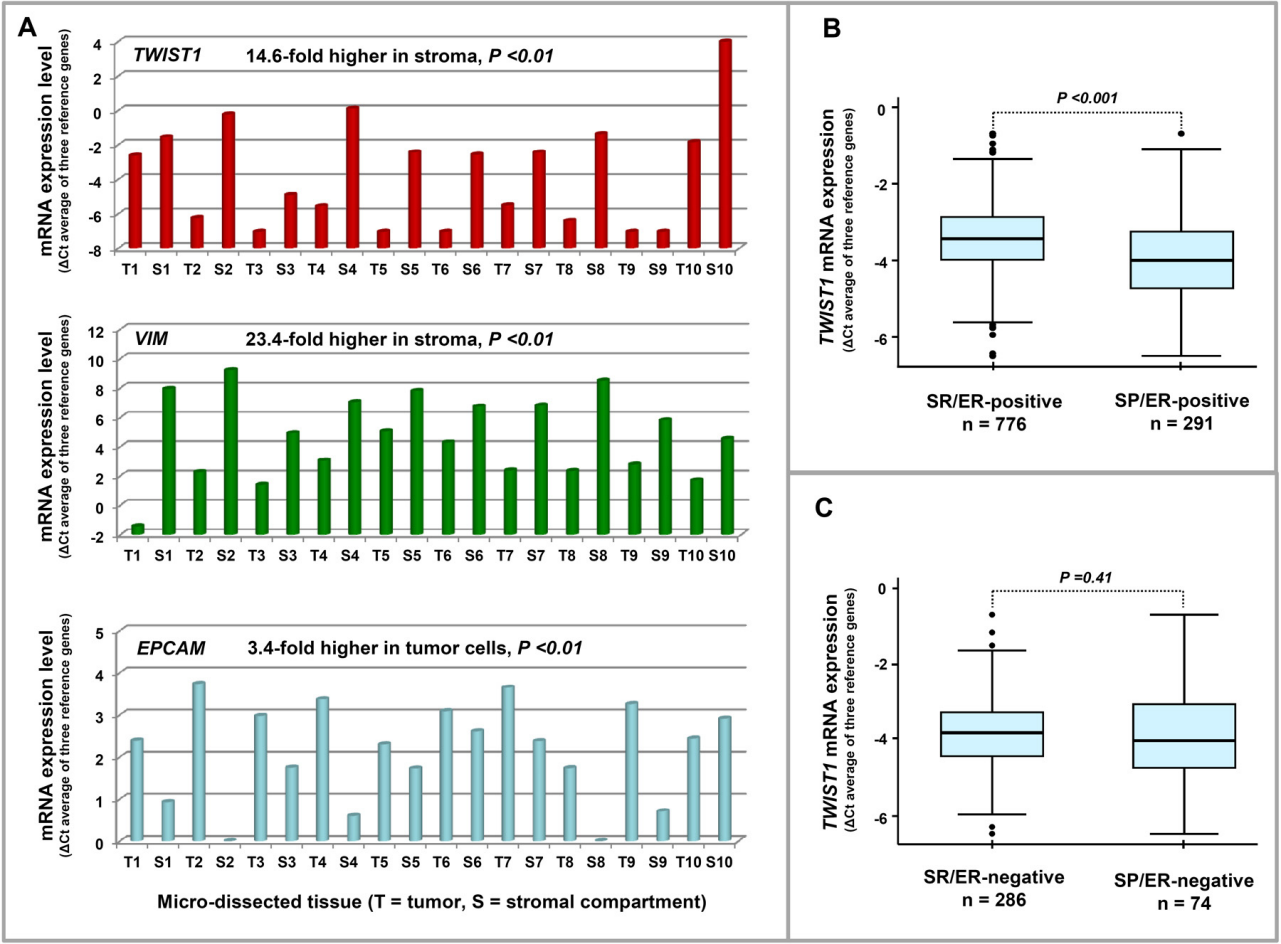
**Association of *TWIST1 *mRNA expression with stromal content of the tumor tissue**. **A: ***TWIST1 *mRNA expression levels in laser capture microdissected breast tumor tissues. The stromal and epithelial tumor cell areas were separately analyzed after microdissecting 10 breast tumor tissues (n = 9 with high *ESR1*expression, n = 1, (T6), with low *ESR1 *expression). After LCM, RNA isolation and cDNA synthesis were done as described in the Patients and Methods sections. *TWIST1 *mRNA levels were measured by qRT-PCR in RNA isolated from both the stromal (S) and the epithelial tumor cell (T) compartments of these tissues, and levels were normalized to our set of reference genes by the delta (Δ) Ct method. To ensure our set of reference genes did not cause a bias in these analyses, and our LCM material was indeed enriched for stromal and epithelial cells, we also measured *VIM *and *EpCAM *in these fractions. A two-sided paired t-test was performed to evaluate the differences. **B: ***TWIST1 *mRNA expression in SR and SP ER-positive tumors and **C: ***TWIST1 *mRNA expression in SR- and SP ER-negative tumors. For figure sections B and C, the mRNA expression of *TWIST1 *was log-transformed and the expression relative to reference genes is presented on the y-axis of the box-and-whisker plots. The box-plot shows the five statistics (box bottom-line is the 25^th ^percentile, solid line in the middle of the box presents the median, upper line of the box is the 75^th ^percentile and the whiskers extend to 1.5 times the inter-quartile range, observations beyond these values are plotted, separately). Mann-Whitney test was performed to evaluate the difference between the SR and the SP groups in both ER-positive and ER-negative patients (B, C).

The co-expression analysis suggested an association between stromal content of the tumor tissue and *TWIST1 *mRNA expression levels. Therefore, we studied the association of *TWIST1 *mRNA expression with tumor stromal content in more detail. For this, we performed LCM to separately isolate the stromal (S) and epithelial tumor cell (T) areas from 10 breast tumor tissues (n = 9 *ESR1 *high, n = 1 *ESR1 *low) with sufficiently micro-dissectible fresh frozen tissue left. After LCM we measured *TWIST1 *mRNA by qRT-PCR in RNA isolated from both the stromal and the epithelial tumor cell fractions of these tissues, and normalized the expression levels to our set of reference genes. To ensure that our reference gene-set did not cause a bias in these analyses, and our LCM material was indeed enriched for stromal and epithelial cells, we also measured *VIM *and *EpCAM *(Epithelial Cell Adhesion Molecule) in these fractions (Figure 3A). In these analyses, *EpCAM *mRNA expression was high in the epithelial tumor cell fractions (3.4-fold, two-sided paired t-test *P *= 0.006) and *VIM *and *TWIST1 *mRNA expression were high in the stromal fractions (23.4-fold, *P *= 0.0001 and 14.6-fold, *P *= 0.0004, respectively). As the prognostic value for *TWIST1 *mRNA expression is confined to ER-positive breast cancer, we assessed whether ER expression might be of relevance for *TWIST1 *mRNA expression in tumor tissues with a variable amount of stromal content. For this, we compared *TWIST1 *mRNA expression levels between stromal rich (SR) and stromal poor (SP) tumors in the ER-positive and ER-negative subgroups. Notably, only in ER-positive tumors, *TWIST1 *mRNA expression was significantly higher in SR compared with SP tumors (Figure 3B, P <0.001); in ER-negative tumors the difference was not significant (Figure 3C, P = 0.41).

**Figure 4:**
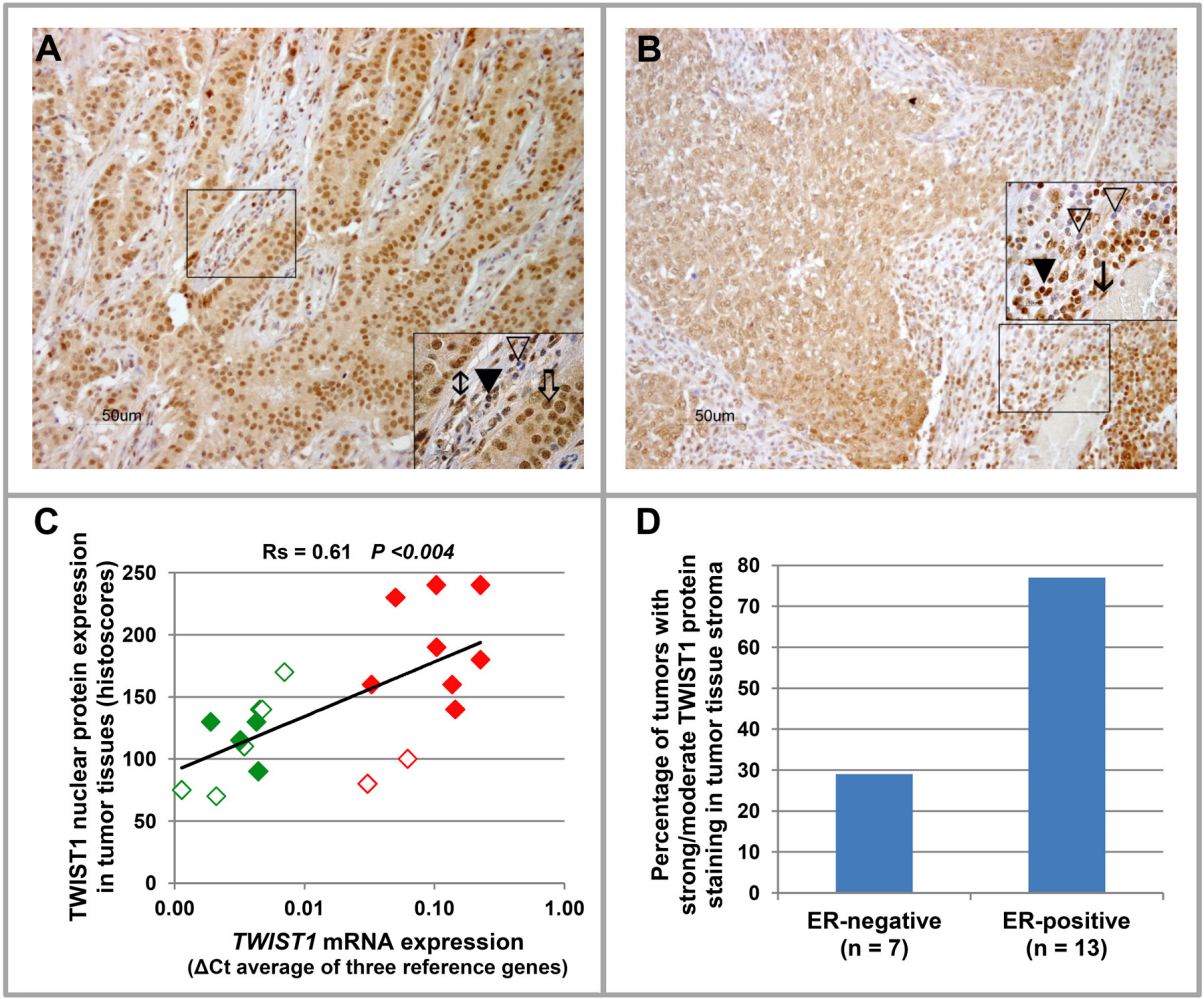
**Representative tissues with IHC staining of TWIST1 protein**. **A: **Strong nuclear staining of TWIST1 protein in an ER-positive tumor tissue with high *TWIST1 *mRNA expression. **B: **Weak nuclear staining of TWIST1 protein in an ER-negative tumor tissue with low *TWIST1 *mRNA expression. **C: **Correlation of *TWIST1 *mRNA expression and protein expression in breast tumor tissues (n = 20; n = 10 high *TWIST1 *mRNA expression, n = 10 low *TWIST1 *mRNA expression). Red and green boxes represent high and low *TWIST1 *mRNA expressing tumor tissues, respectively, whereas empty boxes and filled boxes represent ER-negative and ER-positive tumor tissues, respectively. Spearman rank correlation test was used to evaluate the correlation between *TWIST1 *mRNA and protein expression. **D: **Association of stromal staining of TWIST1protein with ER-positive tumor tissues. (▽ = negative nuclei of inflammatory cells; ▼ = strong positive nuclei of inflammatory cells; ↕ = strong positive nuclei of fibroblast; ⇨ = strong positive nuclei of tumor cells; ↓ = strong positive nuclei of endothelial cells).

This altogether suggests that *TWIST1 *mRNA is predominantly expressed in the stromal compartment of breast tumor tissues. Besides addressing the issue of where *TWIST1 *mRNA is predominantly expressed in breast cancer tissues as described above, we also performed IHC staining to ascertain the localization of TWIST1 protein. For this, we stained 20 tumor tissues with different *TWIST1 *mRNA expression (n = 10 with high *TWIST1 *mRNA expression, n = 10 with low *TWIST1 *mRNA expression). Apparently, TWIST1 protein is predominantly localized in the nuclei of both epithelial tumor cells and in almost all non-epithelial cells present in the stromal compartment of tissues, such as fibroblast cells, endothelial cells and inflammatory cells (Figure 4A, B). In these 20 tumors, *TWIST1 *mRNA expression levels were significantly correlated with the nuclear protein expression in tumor tissue (Spearman rank correlation, Rs = 0.61, *P *<0.004) (Figure 4C). Additionally, tumor tissues showing weak/moderate TWIST1 protein staining were mostly ER-negative (Figure 4C).

Our data suggest that stroma of ER-positive (and not ER-negative tumors) is enriched for TWIST1. To address this in more detail, we stained 20 primary breast tumor tissues (n = 7 ER-negative and n = 13 ER-positive) for TWIST1 protein. We observed that 2 out of 7 (29%) of the ER-negative tumor tissues and 10 out of 13 (77%) of the ER-positive tumor tissues showed moderate to strong TWIST1 protein staining in the stroma. The staining of TWIST1 in the other tumors (71% of the ER-negatives and 23% of the ER-positives) was weak. These data based on a limited number of samples further support our qRT-PCR based findings at the protein level that the stroma of ER positive tumors is enriched for TWIST1 expression (Figure 4D).

## Discussion

The bHLH transcription factor TWIST1, which is essential in developmental processes, such as gastrulation, has been shown to be oncogenic in various cancers [26-33]. Functional studies pin-pointed a pivotal role for this protein in EMT, a fundamental biological process considered to be crucial for metastatic spread in various carcinomas, including breast cancer. This prompted us to analyze mRNA expression of this gene in primary breast tumors from a large retrospective cohort of patients with complete clinical follow-up and address two basic questions. First, can *TWIST1 *mRNA expression in breast cancer tissue predict disease progression? Second, as we had Affymetrix U133A gene-chips data available for a subset of samples [10,11,22], can we reveal which biological pathways are co-expressed with *TWIST1 *in breast cancer?

Our current study shows that high *TWIST1 *mRNA expression is an independent marker of poor outcome in breast cancer patients. As *TWIST1 *mRNA expression was also a predictor of poor outcome in patients with LNN disease who did not receive any adjuvant systemic treatment, we may conclude that *TWIST1 *mRNA expression is a pure prognostic marker and is associated with the natural course of disease progression. This suggests that tumors with high expression of *TWIST1 *are more aggressive, a finding that fits with experimental data where overexpression of *TWIST1 *resulted in increased extravasation during the dissemination of tumor cells [7]. Also in line with this is the increased lymph node involvement of breast tumors exhibiting higher *TWIST1 *expression as observed by us and others [34]. The first seminal paper on *TWIST1 *in breast cancer also showed higher *TWIST1 *mRNA expression in infiltrative lobular carcinoma [7], which we also confirmed, though the implications of this remain unclear.

What we further unexpectedly observed was that the association of *TWIST1 *expression with poor prognosis was confined to ER-positive cases and not seen in ER-negative breast cancer. Similar observations were recently made by Van Nes and colleagues [21]. This group, by studying *TWIST1 *expression in a smaller-sized cohort using IHC on tissue microarrays, also found significantly more cumulative relapses in the ER-positive subgroup of tumors. This observation was not significant in their entire cohort, which also included ER-negative cases. Using the same antibody, we observed, in a subset of formalin-fixed specimen available from our cohort, clear nuclear TWIST1 expression. This protein expression was not only confined to the nuclei of invasive tumor cells but also present in nuclei of other cells, such as fat cells, fibroblasts, endothelial cells and inflammatory cells. Furthermore, although based on small numbers, TWIST1 protein staining was high in those specimens in which we had measured high *TWIST1 *mRNA expression in a matched fresh-frozen sample of the same tumor, while in specimens with low *TWIST1 *mRNA expression the protein staining was overall weaker or absent.

To study the potential biological mechanisms in disease progression connected to *TWIST1 *expression, we identified genes co-expressed with *TWIST1 *in LNN/ER-positive tumors from which genome wide gene expression data were available. Three related pathways were revealed. The first pathway was "Focal adhesion", which involves interaction of ECM with integrin-A and -B ligands. The second pathway, "ECM-receptor interaction", is related to ECM and involves collagen, laminin, fibronetic, tenascin and integrin-A and -B proteins. The third pathway is the "TGF-beta signaling". How these pathways, involving well-known cancer-related genes, are connected to *TWIST1 *expression in relation to disease progression in LNN/ER-positive cancer warrants functional validation. A common denominator among these three pathways, however, is their involvement in ECM remodeling. There is a growing body of evidence showing that changes in the tumor microenvironment can promote proliferation of the tumor cells and, therefore, can influence outcome of patients [35], and our data connect this to *TWIST1 *expression. Furthermore, our work also shows that the stroma is a significant source of *TWIST *transcript expression. Indeed, the current data reinforce recent observations made by Roman-Perez and colleagues after evaluating gene expression patterns in 72 breast tissue samples [36]. They identified two distinct subtypes, Active and Inactive, in the cancer-adjacent extra-tumoral microenvironment of breast tissues and demonstrated that *TWIST1 *was highly expressed along with other stromal associated genes, such as *VIM, ADAMTS2, COL4A2, COL4A1, ITGA7 *and *ITGB1*, in the Active subtype. This could suggest a potential involvement of TWIST1 in the activation of stroma in malignant breast tissue. Furthermore, the Active subtype was associated with expression of TGF-beta induced fibroblast activation signatures, and showed a strong association with OS among specifically ER-positive patients. Intriguingly, also in our data set, the association of *TWIST1 *with the TGF-beta signaling pathway appears to be more prominent in ER-positive tumors.

Another recent study also demonstrated that TWIST1 protein can be found in both compartments of malignant breast tissue, with a predominant expression in the stromal compartment [37]. More importantly, they found that, although rare in their hands, nuclear expression of TWIST1 protein in the epithelial tumor cell compartment to be associated with poor prognosis (OS). TWIST1 protein expression in the stromal compartment was not associated with OS, but positively associated with a positive ER or PGR status of the tumors. This mutual dependence of TWIST1 expression on the presence of a notable stromal component and an ER-positive status of the tumor tissue in our and other studies is in line with the observed prognostic value of *TWIST1 *in ER-positive disease only. Although our study is a correlative study and cannot reveal causality, it is intriguing and points to a novel role of *TWIST1 *in breast cancer.

What is surprising about our results, which associate high *TWIST1 *mRNA expression with poor prognosis in ER-positive breast cancer and with ECM and stromal content, is the fact that up until now the process of EMT has been more frequently assigned to ER-negative cancer and was rarely observed in ER-positive breast cancer. Also, previously studied breast cancer cell lines with clear EMT-like features were ER-negative [38]. This left us with an apparent contradiction. We, therefore, specifically explored whether markers of EMT present on gene expression arrays [11] were associated with *TWIST1 *expression. Of the eight markers analyzed, we found Zinc finger E-box-binding homeobox 1 (*ZEB1)*, Zinc finger E-box-binding homeobox 2 *(ZEB2)*, Snail2 (*SNAI2*), Smooth muscle actin, also known as Alpha-actin-2 (*ACTA2*) and *VIM *clearly, positively correlated with *TWIST1 *expression (Rs = 0.34, 0.34, 0.35, 0.42 and 0.28, respectively; all *P*-values <0.001; Additional file 3: Table S15). This does suggest, in line with the recent study of Roman-Perez and colleagues [28] that markers of EMT are co-expressed with *TWIST1*. However, the lack of a clear negative association of *E-cadherin *mRNA (*CDH1*) with *TWIST1 *mRNA expression in our data set could indicate that EMT may not be fully established. Alternatively, and more in line with our findings, is that some markers of EMT, such as Vimentin and Smooth muscle actin, are also expressed in re-activated stroma [39]. Therefore, our findings are a starting point for more functional studies to be certain about TWIST1 possible role in EMT as well as in re-activated stroma in ER-positive breast cancer.

## Conclusions

In summary, we have found that the *TWIST1 *mRNA expression level is associated with small tumor size, invasive lobular carcinoma, stromal-rich tumors and with *PGR *and *ERBB2 *(over)expression. More importantly, *TWIST1 *mRNA expression predicts, independently of the traditional prognostic factors, poor prognosis in patients with primary breast cancer. This association with poor prognosis is particularly observed in ER-positive disease. For this large retrospective study that involved 1,427 breast cancer specimens, we made use of all clinical material available that met the preset criteria described in Figure 1, thus reaching Level of Evidence III (LOE-III). To reach LOE-I or -II, a prospective randomized study or pooled meta-analysis is required. Studying *TWIST1 *co-expressed genes, we found that ECM related pathways as well as stromal content are correlated with *TWIST1 *mRNA expression in LNN/ER-positive breast cancer. Functional studies are, therefore, required to determine the causal relation between these pathways and the prognostic value of *TWIST1 *mRNA expression in ER-positive tumors.

## Abbreviations

ACTA2: alpha-actin-2; bHLH: basic helix-loop-helix; BM2: beta-2-microglobulin; CI: confidence interval; DFS: disease-free survival; ECM: extracellular matrix; EMT: epithelial to mesenchymal transition; EpCAM: epithelial cell adhesion molecule; ER: estrogen receptor; FDR: false discovery rate; PGR: progesterone receptor; ERBB2: erythroblastic leukemia viral oncogene homolog 2; ESR1: estrogen receptor 1; GEO: gene expression omnibus; GO: gene ontology; HER2: human epidermal growth factor receptor 2; HMBS: hydroxymethylbilane synthase; HPRT1: hypoxanthine phosphoribosyltransferase 1; HR: hazard ratio; IDC: invasive ductal carcinoma; IHC: immunohistochemistry; ILC: invasive lobular carcinoma; KEGG: Kyoto Encyclopedia of Genes and Genomes; LCM: laser capture microdissection; LNN: lymph node-negative; LOE: Level of Evidence; MFS: metastasis-free survival; OS: overall survival; PEN: polyethylene naphthalate; Q: quartile; RT-PCR: reverse transcriptase polymerase chain reaction; SP: stromal poor; SR: stromal rich; TWIST1: Twist-related protein 1: also known as a basic helix-loop-helix protein 38 (bHLHa38); VIM: vimentin; ZEB1: zinc finger e-box-binding homeobox 1; ZEB2: zinc finger e-box-binding homeobox 2.

## Competing interests

The authors declare that they have no competing interests.

## Authors' contributions

AMS, JAF and JWMM designed the study. MR, JAF and JWMM wrote the manuscript. AMS performed the mRNA expression studies. MAT performed the immunohistochemistry. MR, AMS, JAF and JWMM analyzed the data. MR, MPL and MS did the statistical data analyses. All authors approved the final version of the manuscript.

## Supplementary Material

Additional file 1 **Figure S1. Log-transformed distribution of *TWIST1 *mRNA expression levels in the entire cohort of patients. Figure S2**. Kaplan-Meier survival curves presenting the association of *TWIST1 *mRNA expression with overall survival in: A: All 1,427 patients; **B: **LNN patients only; **C: **LNN and ER-positive patients; and **D: **LNN and ER-negative patients. The LNN patients in this study did not receive adjuvant systemic therapy. The patients are divided into four quartiles (Q1 (low) to Q4 (high)) based on *TWIST1 *mRNA expression levels. *TWIST1 *expression levels are presented relative to the expression of our set of three reference genes (*B2M, HMBS *and *HPRT1*). Patients at risk at various time points are indicated. Click here for file

Additional file 2 **Tables S1-S8**. **Table S1: **Model for Uni- and Multivariate analysis for MFS in LNN patients (n = 778). **Table S2: **Model for Uni- and Multivariate analysis for MFS in LNN & ER-negative patients (n = 226). **Table S3: **Model for Uni- and Multivariate analysis for overall survival in all patients (n = 1,427). **Table S4: **Model for Uni- and Multivariate analysis for overall survival in all LNN patients (n = 778). **Table S5: **Model for Uni- and Multivariate analysis for overall survival in LNN & ER-positive patients (n = 552). **Table S6: **Model for Uni- and Multivariate analysis for overall survival LNN & ER-negative patients (n = 226). **Table S7: **Model for Uni- and Multivariate analysis for disease-free survival in all patients (n = 1,427). **Table S8: **Model for Uni- and Multivariate analysis for disease-free survival in LNN & ER-positive patients (n = 552). Click here for file

Additional file 3 **Tables S9-S15**. **Table S9A & B: ***TWIST1 *co-expressed genes measured on Affymetrix U133A gene-chips in LNN/ER-positive patients. **Table S10: **Significant pathways predicted from KEGG database in LNN/ER-positive patients. **Table S11: **Gene ontology enrichment terms from *TWIST1 *co-regulated genes based on their biological processes and cellular components. **Table S12A & B: ***TWIST1 *co-expressed genes measured on Affymetrix U133A gene-chips in LNN/ER-negative patients. **Table S13: **Significant pathways predicted from KEGG database in LNN/ER-negative patients. **Table S14: **Significant pathways predicted from KEGG database in all LNN patients (both ER-positive and ER-negative combined). **Table S15: **Correlation of *TWIST1 *expression with known EMT markers measured on Affymetrix array in LNN/ER-positive samples (n = 221). Click here for file
