# Supplementary figures and images for: High TWIST1 mRNA expression is associated with poor prognosis in lymph node-negative and estrogen receptor-positive human breast cancer and is co-expressed with stromal as well as ECM related genes
Source: Breast Cancer Res. 2012 Sep 11;14(5):R123. doi: 10.1186/bcr3317 (PMC4053101; doi:10.1186/bcr3317)

**Figure S1**

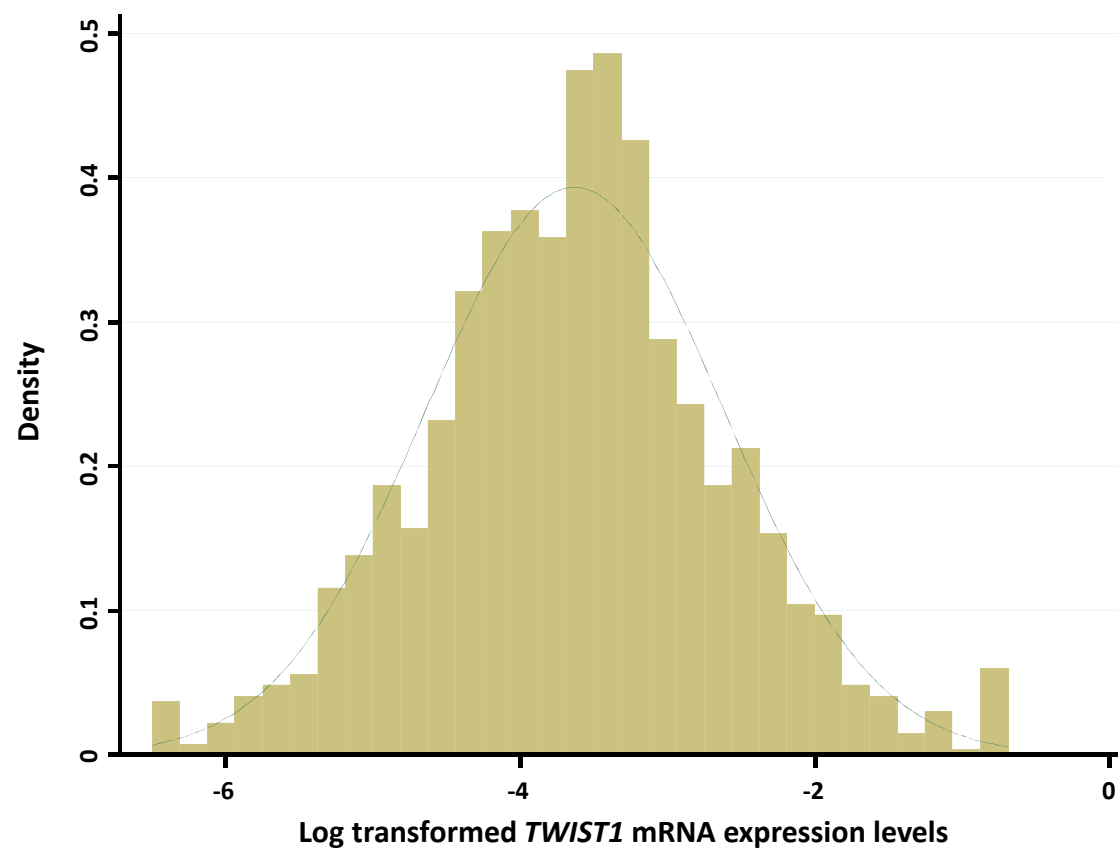

**Figure S2**

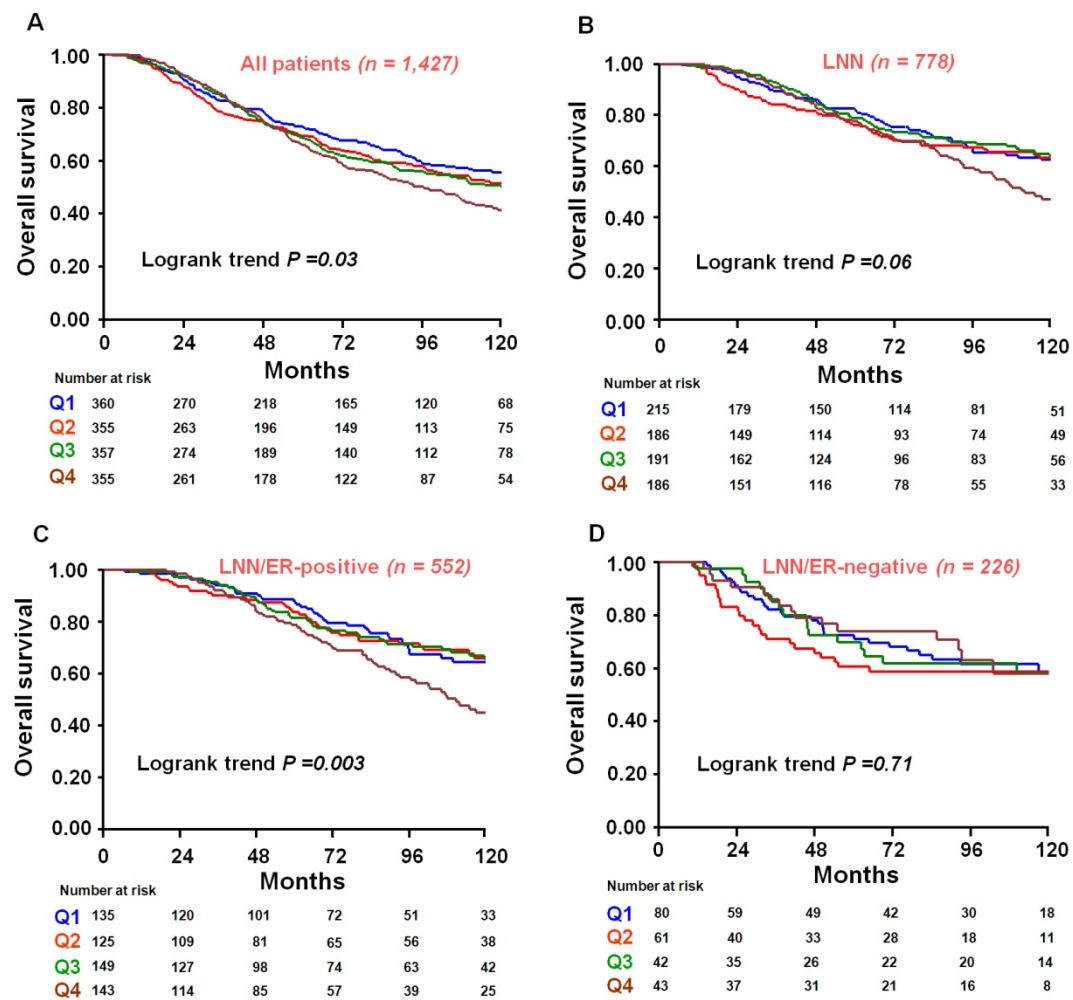

Supplement: Additional file 1 — Figure S1. Log-transformed distribution of TWIST1 mRNA expression levels in the entire cohort of patients. Figure S2. Kaplan-Meier survival curves presenting the association of TWIST1 mRNA expression with overall survival in: A: All 1,427 patients; B: LNN patients only; C: LNN and ER-positive patients; and D: LNN and ER-negative patients. The LNN patients in this study did not receive adjuvant systemic therapy. The patients are divided into four quartiles (Q1 (low) to Q4 (high)) based on TWIST1 mRNA expression levels. TWIST1 expression levels are presented relative to the expression of our set of three reference genes (B2M, HMBS and HPRT1). Patients at risk at various time points are indicated. [file bcr3317-S1.PDF]
