# Supplementary material for: High TWIST1 mRNA expression is associated with poor prognosis in lymph node-negative and estrogen receptor-positive human breast cancer and is co-expressed with stromal as well as ECM related genes
Source: Breast Cancer Res. 2012 Sep 11;14(5):R123. doi: 10.1186/bcr3317 (PMC4053101; doi:10.1186/bcr3317)
Supplement: Additional file 2 — Tables S1-S8. Table S1: Model for Uni- and Multivariate analysis for MFS in LNN patients (n = 778). Table S2: Model for Uni- and Multivariate analysis for MFS in LNN & ER-negative patients (n = 226). Table S3: Model for Uni- and Multivariate analysis for overall survival in all patients (n = 1,427). Table S4: Model for Uni- and Multivariate analysis for overall survival in all LNN patients (n = 778). Table S5: Model for Uni- and Multivariate analysis for overall survival in LNN & ER-positive patients (n = 552). Table S6: Model for Uni- and Multivariate analysis for overall survival LNN & ER-negative patients (n = 226). Table S7: Model for Uni- and Multivariate analysis for disease-free survival in all patients (n = 1,427). Table S8: Model for Uni- and Multivariate analysis for disease-free survival in LNN & ER-positive patients (n = 552). [file bcr3317-S2.PDF]

Table S1: Model for Uni- and Multivariate analysis for MFS in LNN patients (n = 778)

Factors	No. patients	Univariate			Multivariate		
		HR	95% CI	P-values	HR	95% CI	P-values
**Age (years)**							
≤ 40	108	1			1		
41-55	277	0.93	0.66-1.30	0.49	0.98	0.69-1.38	0.90
56-70	249	0.79	0.55-1.12	0.19	0.82	0.47-1.44	0.49
> 70	144	0.57	0.37-0.88	0.01	0.58	0.31-1.09	0.09
**Menopausal status**							
Premenopausal	326	1			1		
Postmenopausal	452	0.78	0.62-0.99	0.04	0.94	0.61-1.46	0.80
**Tumor size**							
pT1,≤ 2 cm	333	1			1		
pT2, > 2-≤ 5	413	1.14	0.90-1.45	0.28	1.14	0.90-1.45	0.28
pT3, > 5 + pT4	32	1.54	0.87-2.73	0.14	1.64	0.92-2.91	0.09
**Grade**							
Poor	402	1			1		
Good/Moderate	147	0.51	0.35-0.73	<0.001	0.53	0.37-0.77	0.001
Unknown	229	1.06	0.82-1.36	0.97	1.12	0.87-1.44	0.38
**ER status** (mRNA)							
Negative	226	1			1		
Positive	552	0.89	0.68-1.14	0.32	1.20	0.87-1.66	0.27
**PgR status** (mRNA)							
Negative	327	1			1		
Positive	451	0.75	0.61-0.94	0.01	0.77	0.57-1.05	0.10
**ERBB2 status** (mRNA)							
Negative	655	1			1		
Positive	123	1.29	0.97-1.73	0.08	1.20	0.89-1.60	0.23
					**Addition to the base model**		
***TWIST1* mRNA level**† (continuous)	778	1.22	1.09-1.36	0.001	1.21	1.07-1.36	0.001
***TWIST1* mRNA level**†							
TWIST1 quartile 1	215	1			1		
TWIST1 quartile 2	186	1.13	0.78-1.54	0.47	1.06	0.74-1.51	0.76
TWIST1 quartile 3	191	1.19	0.86-1.67	0.31	1.10	0.78-1.56	0.60
TWIST1 quartile 4	186	1.65	1.22-2.29	0.002	1.58	1.14-2.20	0.006

TWIST1 mRNA expression level (log-transformed after normalization to the expression of 3 reference genes) was separately added to the base multivariate model that included the factors age, menopausal status, tumor size, grade, lymph-node status, ER, PGR, and ERBB2 mRNA expression levels.

† *TWIST1* mRNA expression level was tested as both continuous variable and in four quartiles

Table S2: Model for Uni- and Multivariate analysis for MFS in LNN & ER-negative patients (n = 226)

Factors	No. patients	Univariate			Multivariate		
		HR	95% CI	P-values	HR	95% CI	P-values
**Age (years)**							
≤ 40	42	1			1		
41-55	90	0.86	0.49-1.50	0.59	0.93	0.51-1.71	0.82
56-70	70	0.81	0.44-1.49	0.5	0.79	0.30-2.07	0.64
> 70	24	0.56	0.22-1.40	0.22	0.52	0.16-1.77	0.3
**Menopausal status**							
Premenopausal	111	1			1		
Postmenopausal	115	0.86	0.56-1.31	0.74	0.93	0.48-2.20	0.93
**Tumor size**							
pT1 ≤ 2 cm	82	1			1		
pT2, > 2-≤ 5	137	1.17	0.75-1.83	0.49	1.14	0.72-1.81	0.6
pT3, > 5 + pT4	7	1.49	0.45-4.89	0.51	1.68	0.49-5.76	0.4
**Grade**							
Poor	145	1			1		
Good/Moderate	23	0.46	0.18-1.14	0.09	0.47	0.19-1.20	0.12
Unknown	58	0.9	0.58-1.52	0.8	0.91	0.56-1.48	0.7
**PgR status** (mRNA)							
Negative	211	1			1		
Positive	15	0.74	0.30-1.82	0.51	0.78	0.31-1.97	0.60
**ERBB2 status** (mRNA)							
Negative	180	1			1		
Positive	46	0.97	0.57-1.61	0.87	0.96	0.56-1.65	0.88
					**Addition to the base model**		
***TWIST1* mRNA level**† (continuous)	226	1.05	0.86-1.29	0.64	1.06	0.85-1.32	0.61
***TWIST1* mRNA level**†							
TWIST1 quartile 1	80	1			1		
TWIST1 quartile 2	61	1.40	0.82-2.40	0.23	1.6	0.90-2.84	0.11
TWIST1 quartile 3	42	1.12	0.62-2.05	0.72	1.23	0.66-2.30	0.51
TWIST1 quartile 4	43	0.99	0.54-1.86	1	1.1	0.58-2.10	0.77

TWIST1 mRNA expression level (log-transformed after normalization to the expression of 3 reference genes) was separately added to the base multivariate model that included the factors age, menopausal status, tumor size, grade, lymph-node status, PGR, and ERBB2 mRNA expression levels.

† *TWIST1* mRNA expression level was tested as both continuous variable and in four quartiles.

Table S3: Model for Uni- and Multivariate analysis for overall survival in all patients (n = 1,427)

Factors	No. patients	Univariate			Multivariate		
		HR	95% CI	P-values	HR	95% CI	P-values
**Age (years)**							
≤ 40	188	1			1		
41-55	530	0.93	0.73-1.19	0.58	0.90	0.70-1.17	0.44
56-70	471	1.09	0.85-1.40	0.47	0.82	0.56-1.19	0.29
> 70	238	1.35	1.02-1.79	0.03	1.1	0.74-1.66	0.6
**Menopausal status**							
Premenopausal	603	1			1		
Postmenopausal	824	1.31	1.12-1.54	0.001	1.34	1.00-1.78	0.05
**Tumor size**							
pT1, ≤ 2 cm	463	1			1		
pT2, > 2-≤ 5	807	1.71	1.43-2.50	<0.001	1.43	1.19-1.73	<0.001
pT3, > 5 + pT4	157	2.87	2.24-3.70	<0.001	1.82	1.40-2.38	<0.001
**Lymph nodes involved**							
0	778	1			1		
1 to 3	288	1.54	1.26-1.88	<0.001	1.60	1.30-1.96	<0.001
>3	361	2.57	2.16-3.06	<0.001	2.34	1.94-2.82	<0.001
**Grade**							
Poor	800	1			1		
Good/Moderate	230	0.62	0.49-0.79	<0.001	0.93	0.77-1.12	0.45
Unknown	397	0.85	0.71-1.01	0.07	0.81	0.64-1.02	0.08
**ER status** (mRNA)							
Negative	360	1			1		
Positive	1,067	0.70	0.59-0.83	<0.001	0.76	0.60-0.95	0.014
**PgR status** (mRNA)							
Negative	572	1			1		
Positive	855	0.64	0.55-0.75	<0.001	0.76	0.62-0.92	0.006
**ERBB2 status** (mRNA)							
Negative	1,187	1			1		
Positive	240	1.39	1.13-1.66	0.001	1.14	0.94-1.39	0.19
					**Addition to the base model**		
***TWIST1* mRNA level**† (continuous)	1,427	1.10	1.08-1.26	0.013	1.11	1.03-1.20	0.006
***TWIST1* mRNA level**†							
TWIST1 quartile 1	369	1			1		
TWIST1 quartile 2	370	1.12	0.89-1.39	0.33	1.14	0.91-1.42	0.26
TWIST1 quartile 3	369	1.12	0.90-1.39	0.32	1.24	0.99-1.55	0.06
TWIST1 quartile 4	368	1.28	1.04-1.59	0.02	1.33	1.06-1.65	0.012

TWIST1 mRNA expression level (log-transformed after normalization to the expression of 3 reference genes) was separately added to the base multivariate model that included the factors age, menopausal status, tumor size, grade, lymph-node status, ER, PGR, and ERBB2 mRNA expression levels.

† *TWIST1* mRNA expression level was tested as both continuous variable and in four quartiles.

Table S4: Model for Uni- and Multivariate analysis for overall survival in all LNN patients (n = 778)

Factors	No. patients	Univariate			Multivariate		
		HR	95% CI	P-values	HR	95% CI	P-values
**Age (years)**							
≤ 40	108	1			1		
41-55	277	0.93	0.65-1.33		0.98	0.67-1.43	0.91
56-70	249	0.99	0.68-1.43		0.93	0.52-1.67	0.81
> 70	144	1.23	0.82-1.84		1.27	0.68-2-.35	0.46
**Menopausal status**							
Premenopausal	326	1			1		
Postmenopausal	452	1.17	0.93-1.48	0.19	1.12	0.71-1.76	0.64
**Tumor size**							
pT1,≤ 2 cm	333	1			1		
pT2, > 2-≤ 5	413	1.22	0.96-1.55	0.11	1.11	0.87-1.43	0.39
pT3, > 5 + pT4	32	1.43	0.79-2.59	0.24	1.40	0.77-2.54	0.28
**Grade**							
Poor	402	1			1		
Good/Moderate	147	0.59	0.42-0.84	0.003	0.99	0.76-1.30	0.97
Unknown	229	0.98	0.76-1.27	0.89	0.66	0.046-0.93	0.21
**ER status** (mRNA)							
Negative	226	1			1		
Positive	552	0.78	0.61-1.00	0.05	1.05	0.76-1.46	0.74
**PgR status** (mRNA)							
Negative	327	1			1		
Positive	451	0.64	0.51-0.80	<0.001	0.65	0.48-0.88	0.006
**ERBB2 status** (mRNA)							
Negative	655	1			1		
Positive	123	1.41	1.06-1.88	0.02	1.33	0.99-1.77	0.06
					**Addition to the base model**		
***TWIST1* mRNA level**† (continuous)	778	1.13	1.01-1.27	0.03	1.16	1.03-1.30	0.01
***TWIST1* mRNA level**†							
TWIST1 quartile 1	215	1			1		
TWIST1 quartile 2	186	1	0.79-1.54	0.56	1.13	0.86-1.59	0.49
TWIST1 quartile 3	191	1.02	0.73-1.42	0.91	1.10	0.78-1.56	0.57
TWIST1 quartile 4	186	1.42	1.03-1.95	0.03	1.52	1.10-2.12	0.01

TWIST1 mRNA expression level (log-transformed after normalization to the expression of 3 reference genes) was separately added to the base multivariate model that included the factors age, menopausal status, tumor size, grade, lymph-node status, ER, PGR, and ERBB2 mRNA expression levels.

† *TWIST1* mRNA expression level was tested as both continuous variable and in four quartiles.

Table S5: Model for Uni- and Multivariate analysis for overall survival in LNN & ER-positive patients (n = 552)

Factors	No. patients	Univariate			Multivariate		
		HR	95% CI	P-values	HR	95% CI	P-values
**Age (years)**							
≤ 40	66	1			1		
41-55	187	1.04	0.65-1.66	0.68	1.04	0.64-1.70	0.86
56-70	179	1.04	0.68-1.43	0.96	0.80	0.38-1.69	0.60
> 70	120	1.40	0.82-1.85	0.31	1.24	0.57-2.70	0.58
**Menopausal status**							
Premenopausal	215	1			1		
Postmenopausal	337	1.20	0.90-1.60	0.22	1.31	0.74-2.35	0.36
**Tumor size**							
pT1, ≤ 2 cm	251	1			1		
pT2, > 2-≤ 5	276	1.14	0.86-1.53	0.11	1.06	0.79-1.42	0.71
pT3, > 5 + pT4	25	1.49	0.79-2.97	0.25	1.68	0.83-3.38	0.15
**Grade**							
Poor	257	1			1		
Good/Moderate	124	0.67	0.46-0.99	0.04	1.18	0.85-1.63	0.33
Unknown	171	1.07	0.78-1.48	0.65	0.75	0.51-1.11	0.16
**PgR status** (mRNA)							
Negative	116	1			1		
Positive	436	0.59	0.43-0.80	0.001	0.64	0.46-0.89	0.009
**ERBB2 status** (mRNA)							
Negative	475	1			1		
Positive	77	1.70	1.20-2.40	0.003	1.60	1.12-2.30	0.01
					**Addition to the base model**		
***TWIST1* mRNA level**† (continuous)	552	1.26	1.09-1.45	0.001	1.27	1.11-1.46	0.001
***TWIST1* mRNA level**†							
TWIST1 quartile 1	135	1			1		
TWIST1 quartile 2	125	1.05	0.67-1.65	0.82	1.08	0.69-1.70	0.73
TWIST1 quartile 3	149	1.15	0.76-1.73	0.52	1.20	0.77-1.86	0.42
TWIST1 quartile 4	143	1.77	1.19-2.63	0.005	1.87	1.24-2.83	0.003

TWIST1 mRNA expression level (log-transformed after normalization to the expression of 3 reference genes) was separately added to the base multivariate model that included the factors age, menopausal status, tumor size, grade, lymph-node status, PGR, and ERBB2 mRNA expression levels.

† *TWIST1* mRNA expression level was tested as both continuous variable and in four quartiles.

Table S6: Model for Uni- and Multivariate analysis for overall survival LNN & ER-negative patients (n = 226)

Factors	No. patients	Univariate			Multivariate		
		HR	95% CI	P-values	HR	95% CI	P-values
**Age (years)**							
≤ 40	42	1			1		
41-55	90	0.79	0.45-1.39	0.50	0.85	0.46-1.56	0.60
56-70	70	0.98	0.54-1.78	0.61	0.87	0.33-2.30	0.91
> 70	24	1.25	0.59-2.65	0.45	1.07	0.36-3.20	0.77
**Menopausal status**							
Premenopausal	111	1			1		
Postmenopausal	115	1.19	0.79-1.80	0.41	1.14	0.53-2.48	0.66
**Tumor size**							
pT1,≤ 2 cm	82	1			1		
pT2, > 2-≤ 5	137	1.32	0.85-2.04	0.22	1.22	0.77-1.93	0.34
pT3, > 5 + pT4	7	1.40	0.43-4.58	0.58	1.32	0.39-4.49	0.66
**Grade**							
Poor	145	1			1		
Good/Moderate	23	0.41	0.17-1.04	0.06	0.47	0.18-1.19	0.11
Unknown	58	0.85	0.53-1.37	0.51	0.84	0.52-1.36	0.48
**PgR status** (mRNA)							
Negative	211	1			1		
Positive	15	0.57	0.21-1.54	0.27	0.66	0.24-1.84	0.43
**ERBB2 status** (mRNA)							
Negative	180	1			1		
Positive	46	0.94	0.57-1.56	0.82		0.59-1.71	0.97
					**Addition to the base model**		
***TWIST1* mRNA level**† (continuous)	226	0.96	0.78-1.18	0.70	0.98	0.79-1.21	0.85
***TWIST1* mRNA level**†							
TWIST1 quartile 1	80	1			1		
TWIST1 quartile 2	61	1.30	0.78-2.17	0.31	1.54	0.89-2.65	0.12
TWIST1 quartile 3	42	0.95	0.52-1.74	0.87	1.03	0.56-1.87	0.93
TWIST1 quartile 4	43	0.93	0.51-1.70	0.82	1.00	0.54-1.87	0.99

TWIST1 mRNA expression level (log-transformed after normalization to the expression of 3 reference genes) was separately added to the base multivariate model that included the factors age, menopausal status, tumor size, grade, lymph-node status, PGR, and ERBB2 mRNA expression levels.

† *TWIST1* mRNA expression level was tested as both continuous variable and in four quartiles.

Table S7: Model for Uni- and Multivariate analysis for disease-free survival in all patients (n = 1,427)

**Factors**	**No. patients**	**Univariate**			**Multivariate**		
		**HR**	**95% CI**	**P-values**	**HR**	**95% CI**	**P-values**
**Age (years)**							
≤ 40	188	1			1		
41-55	530	0.8	0.65-0.98	0.03	0.78	0.63-0.97	0.02
56-70	471	0.85	0.69-1.05	0.13	0.71	0.57-1.21	0.04
> 70	238	0.65	0.51-0.85	0.001	0.56	0.51-0.5	0.002
**Menopausal status**							
Premenopausal	603	1			1		
Postmenopausal	824	0.98	0.86-1.12	0.812	1.24	0.96-1.60	0.09
**Tumor size**							
pT1, ≤ 2 cm	463	1			1		
pT2, > 2-≤ 5	807	1.62	1.38-1.89	<0.001	1.40	1.19-1.64	<0.001
pT3, > 5 + pT4	157	2.44	1.94-3.07	<0.001	1.70	1.33-2.17	<0.001
**Lymph nodes involved**							
0	778	1			1		
1 to 3	288	1.59	1.33-1.89	<0.001	1.50	1.25-1.79	<0.001
>3	361	2.53	2.16-2.96	<0.001	2.27	1.92-2.69	<0.001
**Grade**							
Poor	800	1			1		
Good/Moderate	230	0.61	0.50-0.76	<0.001	0.75	0.61-0.93	0.008
Unknown	397	0.92	0.79-1.08	0.34	1.02	0.87-1.19	0.79
**ER status** (mRNA)							
Negative	360	1			1		
Positive	1,067	0.87	0.74-1.01	0.076	0.89	0.73-1.10	0.278
**PGR status** (mRNA)							
Negative	572	1			1		
Positive	855	0.79	0.68-0.90	<0.001	0.89	0.74-1.07	0.212
**ERBB2 status** (mRNA)							
Negative	1,187	1			1		
Positive	240	1.26	1.06-1.51	0.008	1.16	0.97-1.39	0.1
					**Addition to the base model**		
***TWIST1* mRNA level**† (Continuous)	1,427	1.11	1.04-1.19	0.002	1.11	1.03-1.19	0.005
***TWIST1* mRNA level**†							
TWIST1 quartile 1	360	1			1		
TWIST1 quartile 2	355	1.11	0.91-1.35	0.29	1.04	0.85-1.27	0.695
TWIST1 quartile 3	357	1.11	0.91-1.35	0.29	1.24	0.93-1.39	0.203
TWIST1 quartile 4	355	1.32	1.08-1.59	0.005	1.33	1.02-1.52	0.025

TWIST1 mRNA expression level (log-transformed after normalization to the expression of 3 reference genes) was separately added to the base multivariate model that included the factors such as age, menopausal status, tumor size, grade, ER, PGR, and ERBB2 mRNA expression levels.

† *TWIST1* mRNA expression level was tested as both continuous variable and in four quartiles.

Table S8: Model for Uni- and Multivariate analysis for disease-free survival in LNN & ER-positive patients (n = 552)

**Factors**	**No. patients**	**Univariate**			**Multivariate**		
		**HR**	**95% CI**	**P-values**	**HR**	**95% CI**	**P-values**
**Age (years)**							
≤ 40	66	1			1		
41-55	187	0.72	0.51-1.03	0.07	0.75	0.52-1.10	0.14
56-70	179	0.62	0.43-0.90	0.02	0.62	0.33-1.15	0.13
> 70	120	0.50	0.30-0.73	0.001	0.45	0.23-0.88	0.88
**Menopausal status**							
Premenopaual	215	1			1		
Postmenopausal	337	0.73	0.57-0.93	0.22	1.02	0.62-1.69	0.02
**Tumor size**							
pT1, ≤ 2 cm	251	1			1		
pT2, > 2-≤ 5	276	1.15	0.89-1.47	0.28	1.10	0.85-1.4	0.44
pT3, > 5 + pT4	25	1.51	0.85-2.69	0.15	1.83	1.02-3.28	0.04
**Grade**							
Poor	257	1			1		
Good/Moderate	124	0.68	0.49-0.95	0.02	0.74	0.53-1.03	0.08
Unknown	171	1.07	0.81-1.42	0.59	1.21	0.91-1.60	0.19
**PGR status** (mRNA)							
Negative	116	1			1		
Positive	436	0.69	0.52-0.92	0.01	0.73	0.54-0.98	0.04
**ERBB2 status** (mRNA)							
Negative	475	1			1		
Positive	77	1.41	1.02-1.93	0.036	1.29	0.92-1.78	0.13
					**Addition to the base model**		
***TWIST1* mRNA level**† (Continuous)	552	1.20	1.06-1.35	0.003	1.17	1.03-1.33	0.014
***TWIST1* mRNA level**†							
TWIST1 quartile 1	135	1			1		
TWIST1 quartile 2	125	1.07	0.73-1.55	0.74	0.94	0.64-1.38	0.76
TWIST1 quartile 3	149	1.14	0.80-1.62	0.46	0.97	0.67-1.41	0.89
TWIST1 quartile 4	143	1.64	1.17-2.3	0.004	1.47	1.02-2.09	0.03

TWIST1 mRNA expression level (log-transformed after normalization to the expression of 3 reference genes) was separately added to the base multivariate model that included the factors such as age, menopausal status, tumor size, grade, PGR, and ERBB2 mRNA expression levels.

† *TWIST1* mRNA expression level was tested as both continuous variable and in four quartiles.
